# Supplementary material for: The causal relationship between the human gut microbiota and pyogenic arthritis: a Mendelian randomization study
Source: Front Cell Infect Microbiol. 2024 Nov 26;14:1452480. doi: 10.3389/fcimb.2024.1452480 (PMC11629706; doi:10.3389/fcimb.2024.1452480)
Supplement: Supplementary file 1 [file DataSheet1.zip › mendelian test/ebi-a-GCST90027624.csv_forest.pdf]

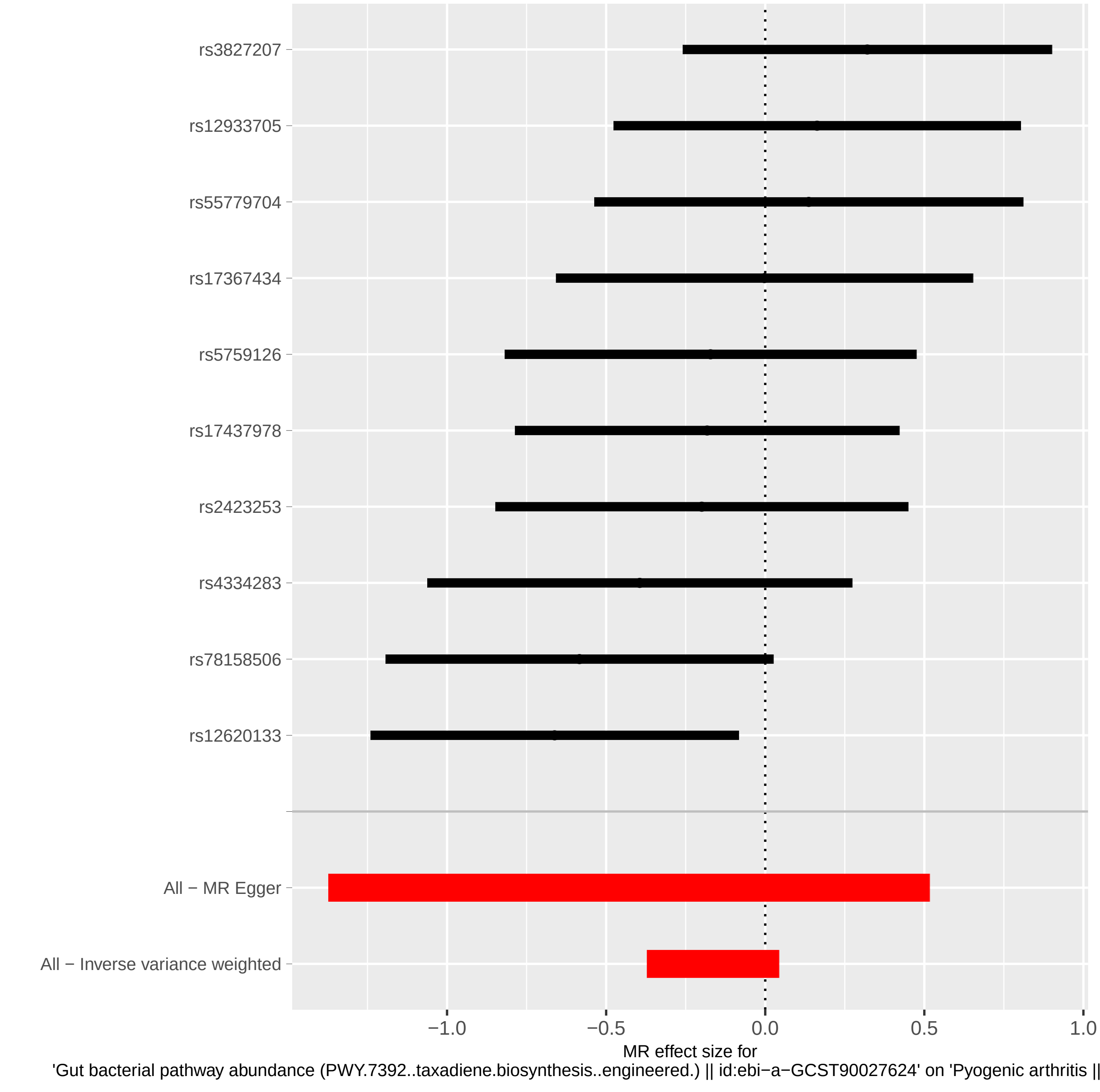

rs3827207
rs12933705
rs55779704
rs17367434
rs5759126
rs17437978
rs2423253
rs4334283
rs78158506
rs12620133
All − MR Egger
All − Inverse variance weighted
−1.0
−0.5
0.0
0.5
1.0
MR effect size for
'Gut bacterial pathway abundance (PWY.7392..taxadiene.biosynthesis..engineered.) || id:ebi−a−GCST90027624' on 'Pyogenic arthritis ||
